# Local Discriminability Determines the Strength of Holistic Processing for Faces in the Fusiform Face Area

**DOI:** 10.3389/fpsyg.2012.00604

**Published:** 2013-01-08

**Authors:** Valerie Goffaux, Christine Schiltz, Marieke Mur, Rainer Goebel

**Affiliations:** ^1^Department of Cognitive Neuroscience, Faculty of Psychology and Neuroscience, Maastricht UniversityMaastricht, Netherlands; ^2^Educational Measurement and Applied Cognitive Science Unit, Faculty of Language and Literature, Humanities, Arts and Education, University of LuxembourgWalferdange, Luxembourg; ^3^Laboratory of Biological Psychology, Faculty of Psychology and Educational Sciences, Katholieke Universiteit LeuvenLeuven, Belgium; ^4^Medical Research Council Cognition and Brain Sciences UnitCambridge, UK; ^5^Department of Neuroimaging and Neuromodeling, Netherlands Institute for Neuroscience, Institute of the Royal Netherlands Academy of Arts and SciencesAmsterdam, Netherlands

**Keywords:** face perception, interactive, holistic, FFA, feature discriminability

## Abstract

Recent evidence suggests that the Fusiform Face Area (FFA) is not exclusively dedicated to the interactive processing of face features, but also contains neurons sensitive to local features. This suggests the existence of both interactive and local processing modes, consistent with recent behavioral findings that the strength of interactive feature processing (IFP) engages most strongly when similar features need to be disambiguated. Here we address whether the engagement of the FFA into interactive versus featural representational modes is governed by local feature discriminability. We scanned human participants while they matched target features within face pairs, independently of the context of distracter features. IFP was operationalized as the failure to match the target without being distracted by distracter features. Picture-plane inversion was used to disrupt IFP while preserving input properties. We found that FFA activation was comparably strong, irrespective of whether similar target features were embedded in dissimilar contexts(i.e., inducing robust IFP) or dissimilar target features were embedded in the same context (i.e., engaging local processing). Second, inversion decreased FFA activation to faces most robustly when similar target features were embedded in dissimilar contexts, indicating that FFA engages into IFP mainly when features cannot be disambiguated at a local level. Third, by means of Spearman rank correlation tests, we show that the local processing of feature differences in the FFA is supported to a large extent by the Occipital Face Area, the Lateral Occipital Complex, and early visual cortex, suggesting that these regions encode the local aspects of face information. The present findings confirm the co-existence of holistic and featural representations in the FFA. Furthermore, they establish FFA as the main contributor to the featural/holistic representational mode switches determined by local discriminability.

## Introduction

Faces are complex visual stimuli that are especially relevant for social interaction. The ability of most humans to decode faces is impressive when considering their high visual homogeneity and the subtlety of the information conveyed. This is probably one of the reasons why face perception has become a central topic in cognitive neuroscience. Clarifying how the brain represents faces will advance our understanding of complex object recognition in general (Connor, 2010).

Predominant theories of face perception suggest that the fast and efficient identification of faces is supported by holistic mechanisms (Farah et al., 1998). Holistic processing is assumed to be an automatic process by which each face is represented as a whole, with little, or no contribution of local information as provided by the features (e.g., nose, eyes, mouth; Tanaka and Farah, 1993). Empirical support for the holistic nature of face representations comes from the observation that face features seem to be obligatorily processed in an interactive way. Interactive feature processing (IFP) manifests itself as a difficulty to process a given feature without being influenced by the surrounding features (Sergent, 1984; Young et al., 1987). Interestingly, face inversion has been shown to disrupt IFP, making observers better at processing features independently of each other (Rhodes et al., 1993; Farah et al., 1998). Since inversion impairs the perception of faces disproportionately compared to other categories (Robbins and McKone, 2007), IFP is thought to be uniquely engaged for faces.

Further confirming the core importance of IFP for faces, neuroimaging evidence indicates that IFP is implemented in the Fusiform Face Area (FFA; Schiltz and Rossion, 2006; Andrews et al., 2010; Schiltz et al., 2010), which is a central region in the face-selective cortical network (Kanwisher et al., 1997; Grill-Spector et al., 2004; Mazard et al., 2006).

We will use the term “interactive” to refer to the empirical evidence that features are processed interdependently. The term “holistic” will be used to refer to the theoretical framework that faces are represented as wholes. Of course these terms are linked as holistic theory is supported by evidence that faces are processed interactively. Other accounts of IFP have however been proposed; it has for example been suggested that IFP arises because humans are particularly sensitive to metric relations between features (for a review, see Maurer et al., 2002). Alternatively it was proposed that both features and their metric relationships are glued into a holistic representation (Tanaka and Sengco, 1997; McKone and Yovel, 2009).

While interactive processing is often highlighted as a unique and automatic face-specific mechanism, the perception of upright faces has also been shown to rely on the local (i.e., independent) processing of features (e.g., Matthews, 1978; Sergent, 1984; Cabeza and Kato, 2000; Leder and Carbon, 2005; Hayward et al., 2008). Until recently, the factors determining the engagement of interactive versus local processing modes were largely unknown. In a recent behavioral study (Goffaux, 2012), we showed that feature discriminability is one of the factors determining whether a given face is processed interactively or locally. Participants were presented with pairs of face pictures and asked to match a target set of features (eyes and brows) independently of the context created by the distracter features (nose and mouth). The strength of IFP was estimated by comparing target matching performance when the target was embedded in a congruent (i.e., “same” targets combined with “same” distracters and “different” targets combined with “different” distracters) or incongruent (i.e., “same” targets combined with “different” distracters and “different” targets combined with “same” distracters) context of distracter features (see also Richler et al., 2008; Goffaux, 2009; Anaki et al., 2011). In contrast to previous studies, we varied the discriminability of the target parametrically. Paired target features could vary by 0% (“same”), 30, 60, or 90% on a morphing continuum. We observed that the size of the congruency effect decayed monotonically as a function of the dissimilarity of the targets within a pair. In other words, the more similar the target features, the stronger the IFP. In contrast, when a clear local feature difference was detected, perceptual contamination by the surrounding distracter features was prevented and IFP was attenuated, or even eliminated.

These findings suggest that IFP is not an all-or-none mechanism automatically engaging for upright faces as suggested by the holistic theory of face perception. Rather, the engagement of IFP in upright faces seems to be determined by the discriminability of the local feature cues relevant for the task. The suggestion that face perception relies on a flexible interplay between interactive and featural modes of processing fits well with recent electrophysiological and fMRI evidence in monkeys and humans showing that FFA is not exclusively dedicated to the interactive encoding of face information, but also contains neurons sensitive to individual feature properties (Yovel and Kanwisher, 2004; Harris and Aguirre, 2008, 2010; Freiwald et al., 2009; James et al., 2010).

The present fMRI study addressed whether the engagement of the FFA into interactive versus featural representational modes is determined by the discriminability of local features, as it is the case for behavioral IFP. We scanned human participants while they performed a discrimination task, in which they had to match target features (eyes and brows) independently of the context of distracter features (nose and mouth; see Goffaux, 2009, 2012). Faces were presented at upright and inverted orientation. We addressed our research question following several lines of exploration.

First, we investigated the amount of IFP engaged in FFA by running an ANOVA with orientation, congruency, and target similarity as factors. If the FFA encodes features interactively at upright orientation mainly when they lack discriminability (i.e., in the incongruent-same condition), we expected that to manifest as a triple interaction between these factors. The triple interaction in the FFA is expected to reflect the largest inversion effect (IE) occurring in the incongruent-same condition.

Second, we investigated IFP in FFA further by measuring the sensitivity of this region to visual differences within face pairs by taking advantage of fMR adaptation (Grill-Spector and Malach, 2001; Grill-Spector et al., 2006). We compared FFA responses to incongruent-different, incongruent-same (i.e., when only a subset of the features differed in a pair), and congruent-different conditions (i.e., when all features differed across faces) to the congruent-same condition (i.e., where the two faces in a pair were identical). If FFA processes feature variations interactively in upright faces one expects that its response does not scale with the number of differing features in a pair (see Harris and Aguirre, 2010; Schiltz et al., 2010). Therefore, there should be no difference in the amount of adaptation release across incongruent-different, incongruent-same, and congruent-different conditions at upright orientation. Based on Goffaux (2012), we know that IFP is recruited when target features in a face pair lack discriminability whereas the detection of a local target difference engenders more local representations. Therefore we hypothesized that if FFA engages both into interactive and featural encoding, its BOLD response to incongruent-same and incongruent-different conditions should be comparable. Since inversion decreases FFA selectivity for face variations (Mazard et al., 2006; Gilaie-Dotan et al., 2010), we expected inversion to eliminate or largely reduce releases from adaptation observed at upright orientation (i.e., no difference between congruent-different, incongruent-different, and incongruent-same conditions on the one hand and congruent-same condition on the other hand). The elimination of adaptation release with inversion would further warrant that the releases observed at upright orientation reflect observer-dependent extraction processes, rather than physical stimulus properties.

Besides the FFA, we explored the neuronal activity profile of the Occipital Face Area (OFA), another face-selective region located in the occipital lobe. OFA was initially proposed to represent features locally before they are glued into a holistic representation by the FFA (Haxby et al., 2000; Liu et al., 2010; Arcurio et al., 2012). The involvement of OFA in local representations of features is further supported by a transcranial magnetic stimulation study where OFA disruption was found to selectively impair the perception of local feature properties (Pitcher et al., 2007). However, some fMRI studies have shown that OFA also codes face features interactively (Schiltz and Rossion, 2006; Goffaux et al., 2009). Moreover, selective damage to OFA has been shown to severely impair face recognition in general (Rossion et al., 2003; Steeves et al., 2006), suggesting that it has a fundamental role in the (holistic) representation of face information. In the present experiment, we intended to further clarify the contribution of OFA to local and interactive aspects of face processing.

We also localized the lateral occipital region (LOC) selective for complex shapes, and the voxels activated by the face stimuli in the early visual cortex (EVC). There are strikingly few neuroimaging studies that investigated IFP in these regions. An exception is the study by Betts and Wilson (2010) who reported no adaptation to local or global feature changes in EVC. Two studies by the same group of authors (Schiltz and Rossion, 2006; Schiltz et al., 2010) used whole-brain analysis to reveal cortical regions outside the face-selective cortical network that may potentially contribute to IFP. However, the poor statistical power afforded by whole-brain analysis may have hindered revealing these contributions. To our knowledge, neuroimaging studies on IFP focused on face-selective regions, and sometimes even exclusively on FFA (Harris and Aguirre, 2008, 2010; James et al., 2010; Liu et al., 2010; Arcurio et al., 2012). Investigating IFP in individually defined visual regions outside the face-selective network was therefore another important aim of the present study. Previous evidence of adaptation release to local and more global feature variations in FFA (and OFA) may indeed be inherited from adaptation in these more general-purposed cortical regions (Mur et al., 2010).

Finally we investigated the functional relationships between these visual regions separately during the interactive and local encoding of face information. Past studies have suggested the FFA contains both holistic and featural representations (e.g., Harris and Aguirre, 2010; James et al., 2010), it could well be that other regions contribute to the flexible switch between holistic and featural representations of face information in this region. We addressed this question based on inter-regions correlation analyses.

## Materials and Methods

### Subjects

Thirteen adult subjects (normal or corrected-to-normal vision; mean age 26 ± 4, 4 males, 2 left-handed; no history of neurological disease) participated in this experiment. They provided their written informed consent prior to participation. They were naïve to the purpose of the experiments. They reported either normal, or corrected-to-normal vision. The experimental protocol was approved by the ethics committee of Maastricht University.

### Stimuli

Grayscale images of Caucasian faces (*n* = 40; half of them male) posing in frontal view and with neutral expression were used. Face images were free of facial hair, glasses, and hairline. Different face images were used in the localizer and congruency experiments. Car images (front view) were used in the localizer experiment. All images were first normalized to obtain a global luminance with zero mean and a standard deviation (i.e., root mean square or RMS contrast) equal to 1 using MatLab 7.5. Images were then filtered using a broadband Gaussian filter (preserving information between 2 and 128 cycles per image, cpi, or 0.34–22 cycles per degree, cpd). The luminance and RMS contrast of each image were adjusted to match the average luminance and contrast of the original image set.

In the localizer experiment, 20 face and 20 car images were presented in intact and scrambled versions. Scrambled images were generated by randomly permuting the phase of the face images in the Fourier domain, a procedure known to preserve SF and orientation content (Dakin et al., 2002; Goffaux et al., 2011). A 3-pixel light gray border surrounded all stimuli.

The congruency experiment required that subjects discriminate faces based only on information within a particular target region while ignoring a complementary distractor region. The target region was located over the eyes and brows and the distractor region over the nose and mouth.

In congruent conditions, both the target and distracter features led to an identical decision. In the congruent-same condition, both target and distracter features were the same across faces in a pair. In the congruent-different condition, they were both different. In incongruent conditions, target and distracter features called for opposite responses. In incongruent-same pairs, face stimuli had identical target but different distracter features. In incongruent-different pairs, face stimuli had different target but identical distracter features. Face contour was stable within all pairs; it only varied across pairs. The present experiment therefore focuses on the interactive processing of inner face features while leaving the potentially important contribution of face contour to IFP (e.g., Andrews et al., 2010) aside. Face gender was also stable within a pair.

**Figure 1:**
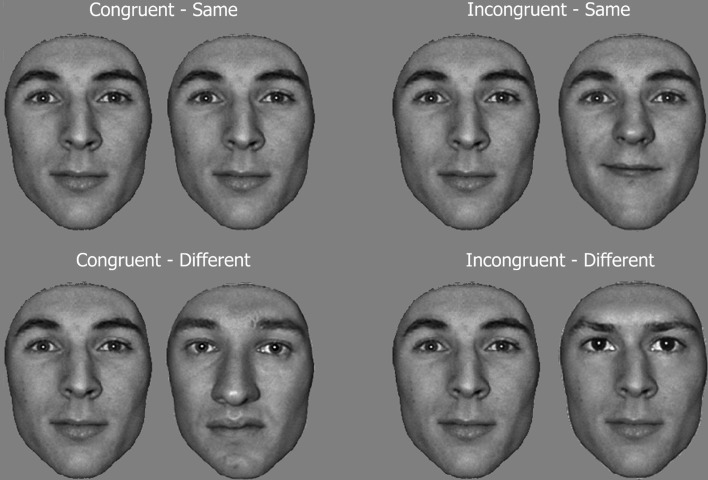
**Example of face pairs in the congruency task**. Subjects had to discriminate a target feature (same/different matching task), i.e., the eyes and eyebrows, while ignoring the context of other features (i.e., distracters: nose and mouth). In congruent conditions, both the target and distracter features lead to an identical decision, while they call for opposite responses in incongruent conditions.

Feature replacement was operated using Adobe Photoshop 7.0. The congruency experiment employed 20 face pictures. Examples of the stimuli are shown in Figure 1.

Visual stimuli were presented using Eprime 1.1 on a uniformly gray background. They were projected onto a translucent screen at the head of the scanner bore by means of a LCD projector and viewed by the subjects through a mirror placed within the RF coil at a viewing distance of 57 cm. Stimulus size was 256 by 256 pixels. At a resolution of 1024 × 768 pixels, all stimuli subtended a visual angle of 5.8° × 5.8°. Behavioral responses were collected during acquisition via a button box.

### Procedure

In the congruency fMRI experiment, faces were presented in pairs and subjects had to report whether the target features (eyes and brows) were same or different across faces by pressing one of two buttons with their right index or middle fingers, irrespective of face context. We used a slow event-related design with picture-plane orientation (upright, inverted), target feature similarity (same, different), and congruency (congruent, incongruent) as within-subject factors. There were 10 trials per condition per run and there were two runs in total, giving a total of 20 trials per condition. Trials (and therefore conditions) were randomly interleaved within a run. The start of a trial was announced by a transiently brighter fixation cross cue (duration: 172 ms). A face then appeared for 200 ms, followed by a 400-ms blank screen. From one trial to the other, the position of the first face was randomly jittered by 10 pixels (0.23° of visual angle) in both *x* and *y* coordinates with respect to screen center. The second face of the pair appeared at the screen center for 400 ms. Spatial jitter prevented subjects from using retinal landmarks while matching face target regions. The presentation of the second face was followed by a long fixation pause (8750 s on average in order to let BOLD response get back to baseline), during which subjects had to report whether the target region (i.e., eyes and brows) differed between the first and second face. Compared to Goffaux (2012), short sequential presentation was preferred in order to prevent eye movements from contaminating the BOLD signal. Several days before the scanning session, subjects were trained with the congruency task on a different set of face stimuli than those used during scanning; training followed the same procedure as described in Goffaux (2012).

Subjects also performed two localizer runs, each comprising 16-s blocks of 20 images: intact faces, intact cars, scrambled faces, or scrambled cars. Within a block, each stimulus appeared during 600 ms at a random *x y* position (±10 pixels away from screen center), followed by a blank screen of 200 ms. During each block, subjects performed a one-back matching task. They were instructed to fixate screen center all along the experiment. Blocks were interleaved with 15 s of fixation pauses. There were three blocks per condition per run.

The localizer experiment, the congruency experiment and a third experiment (reported in Goffaux et al., 2011) were performed on two different days (spread over 2 weeks, on average). The order of experiments and runs was counterbalanced across subjects.

### fMRI acquisition

Imaging was performed on a 3 T head scanner at Maastricht University (Allegra, Siemens Medical Systems, Erlangen, Germany) provided with standard head coil. T2*-weighted echo-planar imaging (EPI) was performed using BOLD contrast as an indirect marker of local neuronal activity.

In the localizer experiment, twenty-five 3.5 mm oblique coronal slices were acquired (no gap, TR = 1500 ms, TE = 28 ms, flip angle = 67°, matrix size = 64 × 64, FOV = 224 mm, in-plane resolution 3.5 mm × 3.5 mm). Each subject performed two localizer runs of 265 TRs each (approximately 400 s).

In the congruency experiment, twenty-one 3.5 mm oblique coronal slices (no gap, TR = 1250 ms, TE = 28 ms, flip angle = 67°; matrix size = 64 × 64, FOV = 224 mm, in-plane resolution 3.5 mm × 3.5 mm) were acquired. Each subject performed two experimental runs, of 665 TRs each (approximately 831 s).

A high-resolution T1-weighted anatomical data set encompassing the whole head was acquired in each session (ADNI sequence, TR = 2250 ms, TE = 26 ms, FA = 9°, matrix size = 256 × 256, FOV = 256 mm^2^, 192 slices, slice thickness = 1 mm, no gap, total run time = 8 min 26 s).

### Behavioral data analysis

Hits and correct rejections in the one-back task of the localizer experiment were combined to compute standard sensitivity estimate (*d*′) individually. *d*′ were then submitted to a repeated-measure ANOVA with category (faces, cars) and stimulus (intact, scrambled) as within-subject factors. These analyses were reported in another paper (Goffaux et al., 2011).

In the congruency experiment, technical problems prevented the recording of the behavioral responses of two subjects. Response accuracy of the remaining 11 subjects was submitted to a 2 × 2 × 2 repeated-measure ANOVA with congruency (congruent, incongruent), target similarity (same, different), and orientation (upright, inverted) as factors. Response times were not analyzed as we did not instruct our subjects to speed their responses.

Conditions were compared two-by-two using Bonferroni *post hoc* tests.

### fMRI data pre-processing

Functional and anatomical images were analyzed using BrainVoyager QX (version 2.1, Brain Innovation, Maastricht, The Netherlands). The first four volumes were skipped to avoid T1 saturation effects. Functional runs then underwent several pre-processing steps: correction of inter-slice scan time differences (using cubic spline interpolation), linear trend removal, temporal high-pass filtering (to remove frequencies lower than three cycles per time course), smoothing with a Gaussian kernel of 6 mm full width at half maximum, and correction for inter-scan head motion (tri-linear-sinc translation and rotation of functional volumes to align them to a reference volume). Anatomical and functional data were spatially normalized to the Talairach coordinate system (Talairach and Tournoux, 1988) with a resolution of 3 mm × 3 mm × 3 mm using sinc interpolation.

### ROI definition

Individual regions of interest (ROIs) were isolated based on the two localizer runs. The localizer runs of each subject were analyzed using an individual fixed effect (FFX) general linear model (GLM). The predictor time courses for stimulation blocks were constructed as box-car functions filtered through a linear model indirectly relating neural activity and BOLD response (Boynton et al., 1996). The predictor time course encompassed the whole trial starting from warning cue onset to the offset of the second stimulus of each pair. We could not separate the contribution of the first and second stimulus to BOLD as the temporal interval separating these events was not long enough or randomly jittered across trials.

For anatomical reference, the statistical maps were overlaid on Talairach-normalized averaged anatomical volumes. The areas that consistently responded preferentially to faces across runs were defined by the conjunction of the contrast [Intact Faces − (Intact Cars + Scrambled Cars + Scrambled Faces)] between the two runs. Significant voxel clusters (at Bonferroni-corrected *p* value < 0.05) on the resulting individual *F* maps were selected as ROIs for further analysis. Face-preferring voxel clusters were located in bilateral middle fusiform gyri (right FFA and left FFA), superior temporal sulci (right STS and left STS), and bilateral inferior occipital gyri (right OFA and left OFA). When one of the ROI could not be found in a given subject, the threshold was progressively lowered to *q*(False Discover Rate, FDR) < 0.001, *q*(FDR) < 0.01, then *q*(FDR) < 0.05. We did not lower the threshold any further to warrant that the ROI clusters were reliably face-preferring. Left and right STS were only found in 7 and 9 out of 13 subjects, respectively, resulting in low statistical power in these regions. We did not analyze these ROIs further.

Additionally, we localized ventral LOC in both hemispheres using the contrast (Intact Cars − Scrambled Cars) at a Bonferroni-corrected *p* value < 0.001 (following Goffaux et al., 2011) in each individual. To ascertain that the LOC ROIs did not prefer one category over the other, individual *z*-scored beta weights from right LOC and left LOC were extracted in each condition of the localizer experiment and submitted to a repeated-measure ANOVA with stimulus (intact, scrambled) and category (face, car) as factors. Afterward, *post hoc* Fisher’s least significant difference (LSD) tests were used to compare conditions two-by-two. We found that the intact-scrambled difference was also significant for faces (*p* < 0.0002). Moreover, there was no significant activation difference between intact faces and intact cars (*p* = 0.6). This confirmed that the presently localized bilateral LOC were not category-selective, as previously reported (Grill-Spector et al., 2001; Grossman and Blake, 2002).

Finally, we used both functional and high-resolution anatomical individual data to localize EVC regions in each subject. EVC were first defined anatomically by centering ellipsoids (12 mm × 5 mm × 5 mm) on 11 consecutive points along the calcarine sulcus of each individual (following Mur et al., 2010). The resulting anatomical ROI included V1 and portions of V2 and V3. Within these anatomically defined EVC areas, we then selected the clusters of voxels which responded to central face stimulation based on the conjunction of contrasts [Intact Faces − Fixation] between the two localizer runs at Bonferroni-corrected *p* value < 0.05. We further tested whether EVC voxels were face-selective by extracting individual *z*-scored beta weights in each condition of the localizer experiment and submitting these values to a repeated-measure ANOVA with stimulus (intact, scrambled) and category (face, car) as factors. *Post hoc* Fisher’s LSD tests were used to compare conditions two-by-two. Main effects of stimulus and category were significant [stimulus: *F*(1,12) = 13.8, *p* < 0.003; category: *F*(1,12) = 8.3, *p* < 0.014]. These factors interacted significantly [*F*(1,12) = 9, *p* < 0.01]. Both left and right EVC regions were indeed more largely activated by intact faces than intact cars (*p* < 0.0002); in contrast, there was no activation difference across scrambled categories (*p* = 0.4).

Talairach coordinates of ROIs were consistent with previous studies (see Table 1).

**Table 1:** **Average Talairach coordinates of individual ROIs**.

Talairach coordinates	Mean			Standard deviation			Number of voxels
	*x*	*y*	*z*	*x*	*y*	*z*	
Right FFA	37	−42	−19	2	5	2	856
Left FFA	−38	−45	−18	5	8	2	665
Right OFA	40	−69	−13	4	6	4	715
Left OFA	−38	−71	−14	5	10	6	191
Right LOC	39	−69	−12	3	4	3	1908
Left LOC	−40	−74	−11	4	4	4	535
Right EVC	15	−90	−3	4	2	6	1362
Left EVC	−11	−91	−7	3	3	5	1298

### ROI analysis

We extracted the activity time course in each individual ROI for each condition of the congruency experiment. We averaged the signal time course across trials in each condition and converted these time courses to percent signal change (PSC) relative to fixation baseline activity (baseline interval: 2 TR of fixation prior to cross cue onset). We then automatically extracted the peak value for each participant in each condition in an interval ranging from two to nine TR post-stimulation. This interval encompassed the peak of the BOLD response related to the presentation of the face pairs while taking BOLD onset delay into account.

### Evaluating IFP by three-way ANOVA and neural IE

Peak values of bilateral FFA, OFA, LOC, and EVC ROIs were submitted to a repeated-measure ANOVA with hemisphere (left, right), orientation (upright, inverted), target similarity (same, different), and congruency (congruent, incongruent) as within-subject factors. If a given ROI processed upright faces differentially depending on local target similarity, we expected to observe a significant triple interaction between orientation, target similarity, and congruency. *Post hoc* Fisher LSD tests were used to compare conditions two-by-two.

Picture-plane inversion is well-known to disrupt IFP (see Goffaux, 2009; Goffaux, 2012) while largely preserving input properties (luminance, contrast, SF spectrum). Therefore, the magnitude of the IE was used to estimate IFP in each Congruency by Similarity conditions. The size of the IE was estimated using η^2^. In the FFA, we expected to observe the largest IE in conditions known to induce robust IFP, namely the incongruent-same condition.

### Release of adaptation to feature differences

We further investigated the sensitivity of each ROI to visual differences within face pairs by taking advantage of fMR adaptation. fMR adaptation refers to the fact that neurons attenuate their responses when the stimulus parameter to which they are tuned is repeated (Grill-Spector and Malach, 2001). By manipulating a stimulus parameter of interest and by measuring the extent to which neuronal response is released from adaptation, one can obtain an indirect measure of the sensitivity of the activated neuronal population to this parameter (Tootell et al., 1995; Kourtzi and Kanwisher, 2001; Huk and Heeger, 2002; however, see Sawamura et al., 2006). In our experiment, the congruent-same condition served as the adaptation condition. The neural responses to congruent-different, incongruent-different and incongruent-same conditions were compared to congruent-same condition using *post hoc* Fisher LSD tests. The size of adaptation release (estimated using η^2^) reflected the ROI sensitivity to face feature variations. If a given ROI encodes face features both locally and interactively, we expected to observe comparable levels of adaptation release in the incongruent-same and incongruent-different conditions (i.e., a sub-additive release from adaptation). If the adaptation releases observed at upright orientation reflects observer-dependent extraction processes, and not physical stimulus properties, then they should be eliminated by inverting the face pairs in the picture-plane (Gilaie-Dotan et al., 2010).

We estimated effect size via η^2^ (Rosnow and Rosenthal, 1996) because this measure quantifies the percentage of PSC variance due to a given factor, independently of sample size. The use of η^2^, and of effect size estimates in general, avoids unwarranted computations based on BOLD subtraction or ratio between conditions (see comments on this issue by Baker et al., 2007; Simmons et al., 2007).

### Inter-ROI correlation

Finally, we investigated the functional relationships between the ROIs by means of two-sided Spearman’s rho correlation analyses (H_0_: rho = 0). BOLD peak in bilateral FFA, OFA, LOC, and EVC ROIs in each experimental condition and for each subject separately were entered in the analysis. The conventional 0.05 alpha level was divided by the number of correlation coefficients computed across ROI pairs (eight correlations were computed per ROI pair, making a total of *n* = 24 therefore providing an adjusted alpha level of 0.002).

## Results

### Localizer behavioral performance

One-back sensitivity was high, in all conditions (Intact faces: 3.8 ± 0.18; Intact cars: 3.55 ± 0.23; Scrambled faces: 3.25 ± 0.16; Scrambled cars: 3.08 ± 0.22) but was significantly affected by category [faces versus cars; *F*(1,12) = 10.86, *p* < 0.006, η^2^ = 0.47] and stimulus [intact versus scrambled; *F*(1,12) = 8.39, *p* < 0.01, η^2^ = 0.41] as subjects performed less accurately for cars than faces and for scrambled than intact stimuli. There was no significant difference between face and car conditions when intact and scrambled conditions were considered separately (*p*s > 0.4).

### IFP behavioral evidence

We addressed whether the discriminability of featural differences influences the correlates of IFP in the face-selective cortical network. Notwithstanding the potential interest of intermediate dissimilarity conditions demonstrated in Goffaux (2012), the present study focused on extreme levels of dissimilarity (0% “same” and 100% “different”) conditions to keep fMRI scanning duration in a reasonable range.

**Figure 2:**
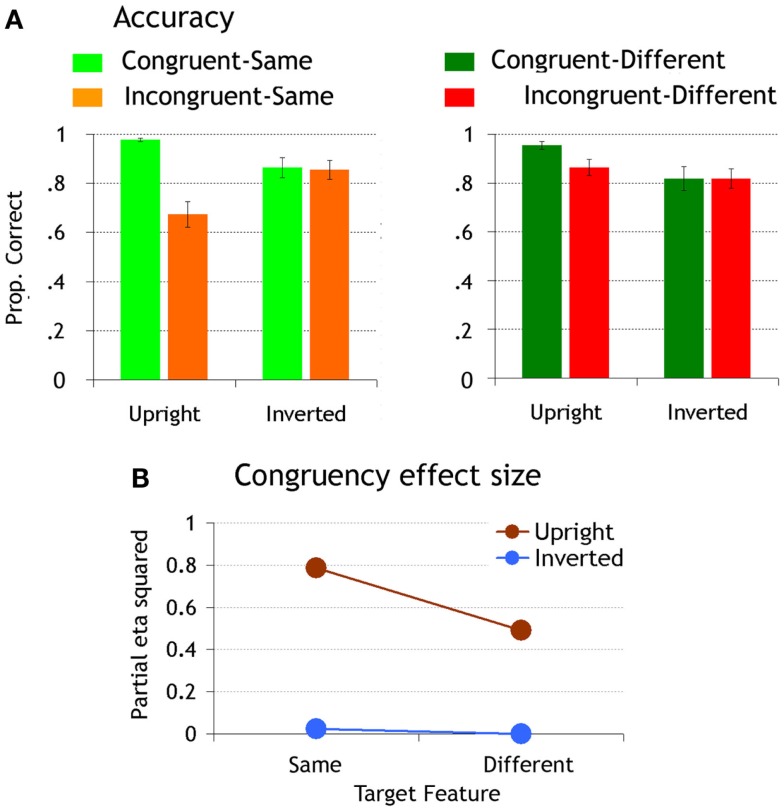
**(A)** Matching accuracy. Error bars represent standard error of the means. **(B)** The size of the congruency effect is plotted for upright and inverted faces as a function of target feature similarity.

In agreement with previous evidence (e.g., Goffaux, 2009, 2012), matching accuracy was worse when the target feature was embedded in an incongruent than a congruent face context [congruency effect: *F*(1,10) = 16.56, *p* < 0.002, η^2^ = 0.62; Figure 2A]. The effect of congruency was moderated by orientation [congruency by orientation interaction: *F*(1,10) = 43.73, *p* < 0.0001, η^2^ = 0.81], and by target similarity [congruency by similarity interaction: *F*(1,10) = 19.36, *p* < 0.001, η^2^ = 0.66]. The triple interaction between these factors was very robust [*F*(1,10) = 39.24, *p* < 0.0001, η^2^ = 0.8].

To study the influence of orientation and target discriminability upon the emergence of IFP, we compared the effect of congruency across orientation by similarity conditions. At upright orientation, the effect of congruency was significant both when target features were same and different (upright-same: *p* < 0.0001, η^2^ = 0.78; upright-different: *p* < 0.007, η^2^ = 0.49); however, in agreement with Goffaux (2012), the congruency effect was far more robust in the “same” (accounting for 79% of accuracy variance) than the “different” conditions (accounting for 49% of accuracy variance; Figure 2B). When faces were inverted, there was no significant congruency effect in any of the conditions (*p*s = 1, η^2^ < 0.02; Figure 2).

We also compared the IE for each congruency by target similarity condition. Inversion decreased accuracy in congruent-same and congruent-different conditions to a comparable extent (congruent-same: *p* < 0.001, η^2^ = 0.39; congruent-different *p* < 0.0002, η^2^ = 0.46). In incongruent conditions, however, it only affected performance when the target features were the same within a pair (incongruent-same: *p* < 0.0001, η^2^ = 0.47; incongruent-different *p* = 0.54, η^2^ = 0.16). Inversion increased performance in this condition as it released the interference from incongruent distracters.

To summarize, behavioral performance in the scanner confirmed that IFP, as indexed by the effects of congruency and inversion, is mostly recruited in upright faces when attended features are similar.

### IFP in (non) face-preferring ROIs

**Figure 3:**
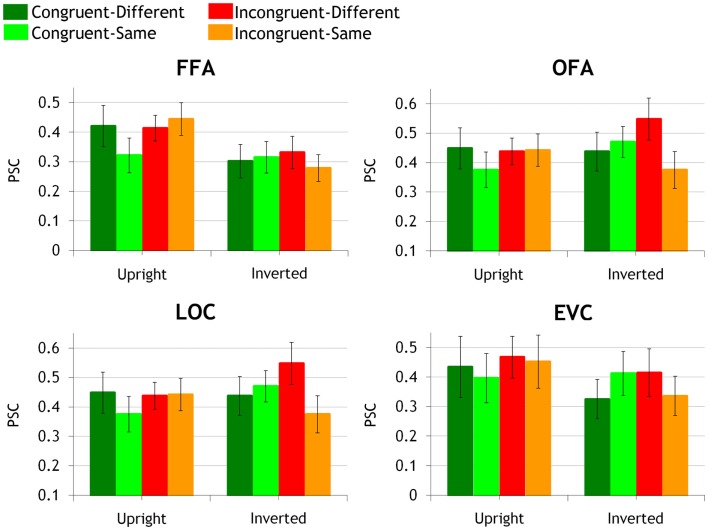
**Activation peak grand averages in bilateral FFA, OFA, LOC, and EVC ROIs are shown in congruent-same, congruent-different, incongruent-same, and incongruent-different conditions, at upright and inverted orientation separately**. Activation peaks (error bars represent mean intra-subject variance) are expressed in percent signal change (PSC) relative to fixation baseline activity (baseline interval: from −2 TR to cue onset).

The BOLD peak values were extracted from the face-preferring ROIs (OFA and FFA) localized *a priori* using two independent localizer runs (see Materials and Methods). Additionally, we localized the non-category-selective LOC and face-selective EVC ROIs (Figure 3). Peak values were first subjected to a three-way ANOVA for repeated measures. Since picture-plane inversion is known to disrupt interactive processing in whole-face displays (e.g., Goffaux, 2009, 2012), the magnitude of the IE was used to estimate IFP in each congruency by similarity conditions. Moreover, we used adaptation effect size to infer the sensitivity of each ROI to interactive versus featural aspects of face information (Grill-Spector and Malach, 2001).

### FFA

#### Evaluating IFP by three-way ANOVA and neural IE

The ANOVA did not disclose any effect or interaction involving the hemispheric factor (*p*s > 0.14). Left and right FFAs are thus jointly considered in the following analyses. In bilateral FFAs, there was a significant main effect of orientation [*F*(1,10) = 16.13, *p* < 0.002, η^2^ = 0.61], with upright faces eliciting larger FFA response than inverted faces. Most importantly, this main effect was qualified by a significant triple interaction between orientation, congruency, and target similarity [*F*(1,10) = 11.25, *p* < 0.007, η^2^ = 0.53].

We explored the triple interaction by comparing the effect of inversion in each congruency by target similarity condition. Inversion significantly decreased neural activity in congruent-different (*p* < 0.0007, η^2^ = 0.35), incongruent-different (*p* < 0.007, η^2^ = 0.29), and incongruent-same condition (*p* < 0.0002, η^2^ = 0.59). Although significant in all conditions, the IE was the most robust in the incongruent-same condition, accounting for approximately 59% of the BOLD peak variance (compared to the 35 and 29% of explained variance in congruent-different and incongruent-different conditions). There was no trend for an IE in congruent-same condition (*p* = 0.62, η^2^ = 0.007) due to fMR adaptation in this condition.

#### Release of adaptation to feature differences

Next, FFA sensitivity properties were investigated by measuring the release from adaptation at upright and inverted orientations separately. At upright orientation, FFA responded with equal strength to congruent-different, incongruent-different, and incongruent-same conditions (*p*s > 0.23; η^2^ < 0.1). Furthermore, each of these conditions induced a similar *amount* of adaptation release, compared to the congruent-same condition (upright-congruent-different: *p* < 0.007, η^2^ = 0.43, upright-incongruent-different: *p* < 0.01, η^2^ = 0.31; upright-incongruent-same: *p* < 0.001, η^2^ = 0.36). When faces were inverted, there was no difference in FFA activation across congruent-different, incongruent-different, incongruent-same, and the adapted congruent-same conditions anymore (*p*s > 0.26, η^2^ < 0.05).

### OFA

#### Evaluating IFP by three-way ANOVA and neural IE

The ANOVA did not disclose any effect or interaction involving the hemispheric factor (*p*s > 0.16). Left and right OFAs are thus jointly considered in the following analyses. The only significant result in bilateral OFAs was the significant triple interaction between orientation, congruency, and target similarity [*F*(1,9) = 11.45, *p* < 0.008, η^2^ = 0.56].

Inversion marginally but non-negligibly *increased* activity in incongruent-different condition (*p* = 0.06, η^2^ = 0.24). It did not modulate neural response in the other conditions (*p*s > 0.13, η^2^ < 0.21).

#### Release of adaptation to feature differences

At upright orientation, there was no release from adaptation, in any of the conditions (*p*s > 0.22, η^2^ < 0.08) and no activity difference between the various congruency by target similarity conditions (*p*s > 0.32, η^2^ < 0.07).

When faces were inverted, no significant release from adaptation could be found either. However, there were differences between congruency by target similarity conditions as OFA activity in response to incongruent-different face pairs was significantly larger than to congruent-different (*p* < 0.04, η^2^ = 0.38) and incongruent-same pairs (*p* < 0.004, η^2^ = 0.53). The OFA response to incongruent-same face pairs was also significantly smaller than to congruent-same pairs (*p* < 0.01, η^2^ = 0.18). There was no difference between inverted congruent-different and incongruent-same conditions (*p* = 0.18, η^2^ = 0.1), and no difference between inverted incongruent-different and congruent-same conditions (*p* = 0.5, η^2^ = 0.009).

### LOC

#### Evaluating IFP by three-way ANOVA and neural IE

The ANOVA revealed a significant effect of hemisphere [*F*(1,12) = 5.07, *p* < 0.044, η^2^ = 0.3] as the response to face pairs was larger in the left- compared to the right-lateralized LOC. The triple interaction between orientation, congruency, and similarity was significant [*F*(1,12) = 16.22, *p* < 0.002, η^2^ = 0.57].

We explored the triple interaction by investigating the IE in each congruency by target similarity condition. Inversion significantly *increased* LOC neural activity in incongruent-different condition only (congruent-same: *p* = 0.15, η^2^ = 0.16; congruent-different: *p* = 0.85, η^2^ = 0.003; incongruent-different: *p* < 0.026, η^2^ = 0.35; incongruent-same: *p* = 0.14, η^2^ = 0.17).

#### Release of adaptation to feature differences

At upright orientation, there was no significant adaptation release; however, adaptation release was non-negligible in congruent-different and incongruent-different conditions (congruent-different: *p* = 0.06, η^2^ = 0.2; incongruent-different: *p* = 0.1, η^2^ = 0.2); it was weaker but still of a non-negligible size in the incongruent-same condition (*p* = 0.08, η^2^ = 0.13).

At inverted orientation, there was a significant release from adaptation in the incongruent-different condition only (*p* < 0.05, η^2^ = 0.09). Furthermore, incongruent-different face pairs induced significantly stronger neural response than congruent-different (*p* < 0.008, η^2^ = 0.44) and incongruent-same conditions (*p* < 0.0003, η^2^ = 0.6). Neural activity in the incongruent-same condition was of significantly smaller amplitude than in congruent-same condition (*p*s < 0.02, η^2^ = 0.16). The LOC response to incongruent-same and congruent-different conditions did not significantly differ (*p* = 0.1, η^2^ = 0.14).

### EVC

#### Evaluating IFP by three-way ANOVA and neural IE

In EVC, there was a significant main effect of hemisphere [*F*(1,12) = 8.44, *p* < 0.01, η^2^ = 0.41], as activation to central face stimuli was larger in left EVC than right EVC. The double interaction between congruency and hemisphere was also significant [*F*(1,12) = 6.02, *p* < 0.03, η^2^ = 0.33]. Incongruent face pairs induced larger neural responses than congruent face pairs in the left EVC only (*p* < 0.0008; right EVC: *p* = 0.4).

#### Release of adaptation to feature differences

There was no adaptation release neither at upright or inverted orientations (*p*s > 0.14, η^2^ < 0.19).

### Inter-ROI correlation

We observed the most robust neural IE in the FFA in the incongruent-same condition, i.e., when local features did not provide any discriminative signal. When target features were discriminable (in congruent-different and incongruent-different conditions), we observed weaker IE, suggesting that faces were then encoded more locally in FFA.

We did not observe this pattern in any other region under study, indicating that FFA is the main contributor to the featural/holistic weighting in representational modes depending on local discriminability. However, this does not preclude that the other investigated ROIs contribute to the FFA activation profile.

We were therefore interested to investigate whether OFA, LOC, and EVC might contribute to local featural processing in FFA. To answer that question, we calculated the inter-region correlations in each experimental condition separately by means of two-sided Spearman’s rank correlation tests. We expected the strongest functional relationship between FFA and the other ROIs when local processing is engaged most strongly (i.e., in inverted conditions in general and in both upright and inverted incongruent-different conditions).

**Figure 4:**
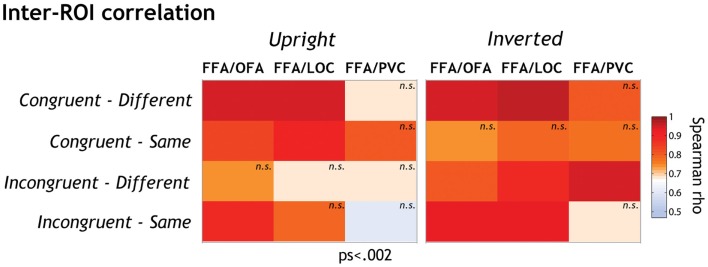
**The functional relationships between FFA on the one side and the other ROIs (OFA, LOC, and EVC) on the other side were explored by means of two-sided Spearman’s rho correlation analyses (alpha level corrected for multiple analyses: 0.002)**. Spearman rho coefficients are color-coded for each condition separately. We performed a standard two-sided test on Spearman’s rho to determine whether inter-ROI correlations of activation were significantly different from expected by chance. Correlation coefficients differed significantly from 0 at *p* < 0.002 except when labeled “n.s.”

Spearman’s rank correlation tests revealed a statistically significant and strong relationship between FFA on the one hand and OFA and LOC on the other hand (see Figure 4; Table 2). There were, however, some interesting variations of correlation strength across experimental conditions.

**Table 2:** **Spearman rho coefficients of inter-ROI correlations**.

Inter-ROI correlation	Upright orientation			Inverted orientation		
Spearman rho coefficients	FFA–OFA	FFA–LOC	FFA–EVC	FFA–OFA	FFA–LOC	FFA–EVC
Congruent – different	0.94	0.95	0.67	0.94	0.96	0.81
Congruent – same	0.83	0.9	0.81	0.74	0.78	0.77
Incongruent – different	0.73	0.68	0.69	0.81	0.88	0.94
Incongruent – same	0.88	0.78	0.6	0.93	0.93	0.68

Overall inversion increased FFA-LOC and FFA-OFA correlation in incongruent conditions (accounting on average for 54 and 65% of activation variance, respectively). When faces were inverted, inter-ROI relationships got stronger in these conditions (accounting on average for 82 and 76% of variance, respectively). Given that inversion renders the processing of face information more local, this correlation suggests that LOC and OFA might contribute to FFA featural encoding in a greater extent than to interactive processing.

It is however important to note that FFA-OFA neural responses also significantly correlated in the upright incongruent-same condition, i.e., when interactive processing was most strongly involved. Nevertheless, this correlation got stronger with inversion, indicating that the FFA-OFA relationship supports the local more than the interactive processing of features.

The FFA-LOC and FFA-OFA relationships were not influenced by face orientation in congruent-different face pairs. In the congruent-same condition, the FFA-LOC and FFA-OFA relationships were only significant at upright orientation. As discussed later, the patterns observed in congruent conditions are however difficult to interpret.

The functional link between EVC and FFA only reached significance in the inverted-incongruent-different condition where it accounted for 88% of variance. This result suggests that EVC also contributes to the encoding of local feature differences in inverted faces in FFA.

## Discussion

How does the human brain represent faces? Answering this question will provide invaluable insight on how brain function generates complex visual experiences. The holistic theory of face perception states that faces are automatically represented as wholes, with little, or no contribution of local feature cues. The interactivity of feature processing is taken to support holistic theory. However, growing behavioral evidence indicates that the local information provided by the features also contributes to face processing (e.g., Cabeza and Kato, 2000; Leder and Carbon, 2005). More recent evidence suggests that IFP is not automatic for faces, but engages when local features are difficult to discriminate. In contrast, when features contain discriminative information, IFP disengages in favor of a more local representational mode (Goffaux, 2012).

That face perception relies on a flexible interplay between interactive and featural modes of processing fits with recent electrophysiological and fMRI evidence in monkeys and humans showing that FFA is not exclusively dedicated to the holistic representation of faces, but also contains neurons sensitive to individual features (Yovel and Kanwisher, 2004; Harris and Aguirre, 2008, 2010; Freiwald et al., 2009; James et al., 2010).

The present fMRI study addressed whether the engagement of the FFA into interactive versus featural representational modes is governed by the discriminability of local features as is the case for behavioral IFP. Subjects were scanned while they performed a feature discrimination task in congruent or incongruent face contexts. In such a task, IFP is operationalized as the failure to match a target feature between two faces without being distracted by task-irrelevant surrounding (distracter) features. We made three main observations.

### Adaptation release in response to feature manipulations in the FFA

We inferred the sensitivity of the FFA to the various manipulations of face information based on the well-known phenomenon of release from fMR adaptation (Grill-Spector and Malach, 2001). More specifically, we compared FFA responses to incongruent-different, incongruent-same (i.e., when only a subset of the features differed in a pair), and congruent-different conditions (i.e., when all features differed across faces) to the congruent-same condition (i.e., where the two faces in a pair were identical). We observed that the FFA BOLD response was equally large when all features or only a subset of them differed in a pair of upright faces. This non-linear, also called sub-additive, response of FFA to face feature variation has been taken to support the view that features are not represented independently in this region but are rather glued into a holistic representation (Schiltz and Rossion, 2006; Harris and Aguirre, 2010; Schiltz et al., 2010). Based on the behavioral performance of our present and previous participants (Goffaux, 2012), we can be more specific in our conclusions and report that FFA activation was comparably strong, irrespective of whether feature variations were processed interactively (in the incongruent-same condition) or more locally (in the incongruent-different condition).

By means of a continuous carry-over adaptation design and stimulus morphing technique, Harris and Aguirre (2010) also tested whether FFA adaptation release in response to different amounts of feature variations is additive (as expected in case of independent and local processing of feature variations) or sub-additive (as predicted by IFP). These authors compared a “pure” condition where faces varied by 100% on the morphing continuum at the level of only one feature, to a “composite” condition where two features varied each by 50% on the morphing continuum. Like in the present study, they reported similar amounts of adaptation release across these two conditions in the right FFA, indicating that this region encoded feature variations interactively. In another experiment, the “pure” condition was contrasted to a variant of the “composite” condition, in which one feature varied more extremely than the other manipulated feature (e.g., 87.5% variation of one feature combined with 50% variation of the other feature). In the latter situation, they found that the right FFA activation released from adaptation to the “composite” condition in an additive way. This finding mirrors our behavioral observation that the more discriminable the local features the more locally they are encoded. The present fMRI findings substantiate these previous indications that holistic and featural representations co-exist in the FFA (see also Harris and Aguirre, 2008; Betts and Wilson, 2010; James et al., 2010) and that the flexible switch between interactive and featural encoding in this region is governed by the discriminability of local information provided by the features.

We observed, as others before, that inversion eliminates the fMRI adaptation releases observed for upright faces in the FFA, suggesting that inversion disrupts FFA sensitivity to face variations (e.g., Yovel and Kanwisher, 2005; Mazard et al., 2006; Gilaie-Dotan et al., 2010). FFA activity modulations observed at upright orientation thus have to be attributed to subjective perception since inversion preserves most physical properties of the stimulus (feature configuration, and spectral properties).

### The effect of inversion on FFA interactive and local processing

Since inversion disrupts interactive more than local aspects of face processing (e.g., Goffaux, 2009), we used the neural IE as a measure of IFP involvement in the various congruency by target similarity conditions. We showed that the IE in FFA was most robust in the incongruent-same condition. Inversion decreased the FFA response to the incongruent-different condition as well but its effect on this condition was half the size smaller than on the incongruent-same condition. The fact that inversion mainly disrupted FFA response to incongruent-same stimuli further establishes that IFP engages mainly when local featural signals cannot be disambiguated at the local level (see below for a discussion of Maurer et al., 2007; Rotshtein et al., 2007; Goffaux et al., 2009 findings based on the comparison of featural versus relational manipulations of faces). The disproportionate IE for the incongruent-same condition was not observed in the other ROIs under study, suggesting FFA as the main contributor to the featural/holistic representational mode switches as a function of local discriminability.

Although much weaker than in incongruent-same condition, the effect of inversion was also significant in the other conditions and more surprisingly in the incongruent-different condition assumed to produce local representations. Neuroimaging investigations on the IFP (Betts and Wilson, 2010; Harris and Aguirre, 2010; James et al., 2010) so far failed to investigate how the adaptation releases observed at upright orientation for local feature variations were affected by inversion. In their study on the composite illusion, Schiltz and Rossion (2006) tested the effect of inversion on fMR adaptation, but only in conditions where the target face half was identical (similar to our incongruent-same condition) and not in conditions where the target face half differed. Some hint comes from one of our studies (Goffaux et al., 2009), in which we manipulated faces at the level of local features versus feature relations. We also found that inversion decreased FFA response to local featural changes in left FFA (and marginally in the right FFA) despite the fact that inversion barely affected behavioral performance in this condition.

Based on this evidence we can only speculate that there might exist a default orientation-dependent processing mode in the FFA, which would activate whenever a face stimulus is processed. Such mechanism would operate with a varying intensity depending on the local versus interactive processing mode engaged.

Our conclusions largely rely on observations made in incongruent conditions because congruent conditions were here taken as baseline conditions against which performance in incongruent conditions was compared in order to estimate the engagement of IFP, as conventionally done in behavioral congruency experiments. The neural mechanisms involved in congruent conditions are therefore not totally clear. In the congruent-different condition, observers were presented with a global variation of inner features; the fact that both behavioral and neural IE in congruent-different were of an intermediate size compared to incongruent-different and incongruent-same conditions indicates that congruent-different condition resides between incongruent-same and incongruent-different extremes in terms of the amount of IFP engaged. For the congruent-same condition, BOLD response was driven by fMR adaptation in FFA. However, it is clear that this was not the case in OFA where this condition induced a higher BOLD response than incongruent-same conditions at inverted orientation. This counterintuitive finding needs to be explored further (see also below).

### Adaptation release and inversion effect in the OFA, LOC, and EVC

A striking difference between FFA and OFA processing is that there was not even a trend for adaptation release in OFA at upright orientation. At inverted orientation, however, OFA responded significantly more robustly to incongruent-different than incongruent-same and even congruent-different conditions. The fact that OFA responded more strongly when *only* the target features differed in a pair than when all features differed indicates that this region may be highly specialized in the local processing of face features. Arcurio et al. (2012) recently reported that OFA maximally responded to eye features when presented in isolation. OFA actually *decreased* its response when more differing features (nose and mouth) were added to the display. We suspect that we observed a similar phenomenon here. Namely, the presence of variation in non-preferred features (nose and mouth in inverted congruent-different and incongruent-same conditions) may have inhibited OFA response to the target feature.

Inhibition may also explain why the OFA response for incongruent-same face pairs was weaker than for congruent-same pairs at inverted orientation. Since inversion disrupts IFP, the target feature should actually not suffer from the presence of distracter features in inverted incongruent-same face pairs. Moreover there is good evidence that when faces are inverted human observers become mainly sensitive to restricted regions of the face (e.g., Van Belle et al., 2010). For these reasons we would have expected the inverted incongruent-same and congruent-same conditions to lead to a comparable BOLD response in OFA (as shown by Schiltz and Rossion, 2006; Schiltz et al. 2010) and LOC since in both cases the local target feature is “same.” Further research is needed to explore inhibition during the perception of faces in OFA.

In the non-face-selective LOC, the adaptation releases though marginal at upright orientation were of a non-negligible size. When faces were inverted, LOC responded mostly to local target feature differences like the OFA further confirming the role of LOC in the part-based representation of faces and possibly other visual categories (Yovel and Kanwisher, 2004; Kanwisher and Yovel, 2006).

Besides the high-level visual regions, we also explored voxel clusters responding to faces in the EVC. Activation in the left-lateralized EVC was sensitive to local feature differences relatively independently of face orientation, indicating that FFA sensitivity (which was modulated by inversion) did not merely reflect early visual processing. Although demonstrating some face-selectivity, these regions may thus encode feature variations locally, based on general-purpose mechanisms.

### Contribution from OFA, LOC, and EVC to FFA local feature encoding

By means of Spearman rank correlation tests, we showed that OFA and LOC regions best predicted the FFA activation profile in incongruent conditions at inverted orientation. Since inversion is known to promote local feature encoding, this suggests that the representation of featural differences in the FFA might be supported by OFA and LOC processing. Interestingly, OFA was also found to significantly contribute to FFA activation in upright incongruent-same condition, i.e., when face processing was most interactive. Yet, this correlation got stronger with inversion, further suggesting that FFA-OFA relationship supports the local more than the interactive processing of features. Overall, the inter-ROI correlations indicate that, among the cortical regions analyzed here, the FFA is the main cortical site for IFP.

Spearman analyses further indicated that EVC activation was a reliable predictor of FFA activation only in the condition where the faces were processed most locally due to the combined influence of the presence of local target differences and inversion (i.e., the inverted-incongruent-different condition). The small receptive field size of neurons in EVC may be particularly useful in this condition. In future studies, it would be particularly interesting to explore how the functional connectivity between FFA and early visual regions varies depending on the engagement of local versus interactive modes of face processing.

### Implications for the study of face (holistic) processing

Previous evidence hinted that the strength of IFP may depend on the discriminability of local featural signals. In their influential paper, Farah et al. (1998) used a congruency paradigm similar to ours except that subjects only knew which feature to attend (eyes, nose, or mouth) *after* the presentation of the faces. Interestingly, they also reported, without further discussing, stronger congruency effects when target features were identical than when they differed. More recently, Yovel and Duchaine (2006) reported that the magnitude of face IE, taken as an indicator of IFP strength, decreases when feature differences are made more salient, e.g., by varying not only shape but also the color properties of features. In a systematic review of the literature, McKone and Yovel (2009) showed that the decrease of the IE as a function of feature color/brightness dissimilarity generalizes to various kinds of tasks, including bizarreness ratings, distinctiveness ratings, recognition memory, familiar faces naming and matching. This suggests that IFP dependence on feature discriminability is not restricted to situations where eye region has to be selectively discriminated as in the congruency paradigm employed here but seems to generalize to the processing of other features, to whole-face discrimination and recognition tasks (see also e.g., Cabeza and Kato, 2000; Leder and Carbon, 2005; Busigny et al., 2012).

Support for the holistic theory of face perception largely relies on the composite illusion. The composite illusion refers to the observation that while discriminating features presented in whole-faces, identical features look different when embedded within different whole-face contexts (Hole, 1994). In composite illusion studies, the performance measured when different parts are embedded in identical (i.e., incongruent) contexts is barely considered as it is implicitly assumed that no illusion should arise in this situation. Originally, however, holistic theory states that features are automatically processed in an interactive way, independent of the similarity relationship between facial elements. Because it is limited to the “same” response modality, there has been a recent debate as to whether the composite illusion is a valid measure of holistic processing (Richler et al., 2011). Our past and present results show that holistic processing is mostly engaged in the case of non-discriminable local targets. Therefore, the composite illusion seems to be a valid *marker* of holistic processing. Nevertheless, we think that ignoring the “different” trials, as has been done in most previous composite illusion studies, limits our understanding of holistic face perception as this procedure obscures an important facet of holistic processing, i.e., that its role is to disambiguate local face signals.

Past and present evidence shows that inversion only moderately affects the activation of FFA (and behavioral performance) when local featural differences are to be processed (e.g., Maurer et al., 2007; Rotshtein et al., 2007; Goffaux et al., 2009 but see Yovel and Kanwisher, 2004). We confirm these results here. In contrast, these studies showed that when differences in feature spacing are to be detected, inversion largely affects FFA (and behavioral) response. Here, we show that it is not so much the nature of change applied to the face stimulus (feature replacement or displacement) that predicts the sensitivity of FFA to inversion. Rather, we show that the same manipulation (i.e., replacing local features within a pair of faces) can induce large or no neural IE depending on how this change is processed by the observer; i.e., interactively versus locally. As discussed by others (Goffaux and Rossion, 2007; Rossion, 2008), this aspect has been dismissed, but explains why sometimes a large neural IE has been observed for so-called featural manipulations (cf. Yovel and Kanwisher, 2004). Here we show that featural changes applied to distracter features in a congruency task induce robust IFP.

### Temporal aspects of IFP dependence upon local discriminability

IFP is thought to occur early during the course of visual processing (Jacques and Rossion, 2009). This view is further supported by the observation that IFP is mainly driven by the low spatial frequencies of the face image (Goffaux and Rossion, 2006; Goffaux, 2009), which are themselves encoded early in the face-selective cortical network (Goffaux et al., 2011). Given the poor temporal resolution of the present behavioral and fMRI investigations, future studies should address whether IFP dependence upon feature discriminability occurs in early steps of visual processing or whether it arises in later processing stages.

## Summary and Conclusion

In summary, the present fMRI evidence indicates that the FFA contains both interactive/holistic and featural representations of faces and that this region flexibly switches from one representational mode to the other as a function of the local discriminative information content of faces. When discriminability is low IFP is strongest, whereas highly discriminable features activate more local representations in this region. OFA, LOC, and EVC are suggested to contribute to the local face processing in the FFA, whereas IFP seems to stem predominantly from the FFA.

## Conflict of Interest Statement

The authors declare that the research was conducted in the absence of any commercial or financial relationships that could be construed as a potential conflict of interest.
